# Supplementary material for: Orbital Reconstruction in a Self-assembled Oxygen Vacancy Nanostructure
Source: Sci Rep. 2015 Jul 27;5:12402. doi: 10.1038/srep12402 (PMC4515763; doi:10.1038/srep12402)
Supplement: Supplementary Information [file srep12402-s1.pdf]

Supplementary Information for

Orbital Reconstruction in a Self-assembled Oxygen Vacancy Nanostructure

H. Jang[1], G. Kerr[1], J. S. Lim[2], C.-H. Yang[2], C.-C. Kao[3], and J.-S. Lee[1,*]

[1]*Stanford Synchrotron Radiation Lightsource, SLAC National Accelerator Laboratory, Menlo Park, California 94025, USA*

[2]*Department of Physics, KAIST, Yuseong-gu, Daejeon 305-701, South Korea*

[3]*SLAC National Accelerator Laboratory, Menlo Park, California 94025, USA*

**jslee@slac.stanford.edu*

[S. 1] Manipulation of a periodicity of oxygen vacancy

When Ca^{2+} substitution rate (x) is larger than ~ 0.15, oxygen vacancy superstructure is formed[S1] and the periodicity empirically shows ~ $1.5/x$. When x becomes 0.20, 0.25, and 0.30, the periodicity becomes 8 unit cells (u.c.), 6 u.c., and 5 u.c., respectively and corresponding superstructure reflection positions changed (Fig. S1). When x = 0.25, 2θ angle of second superstructure reflection (002) is near 90° and therefore the structural contributions are suppressed with π polarized incident light (see section [S. 2] for details).

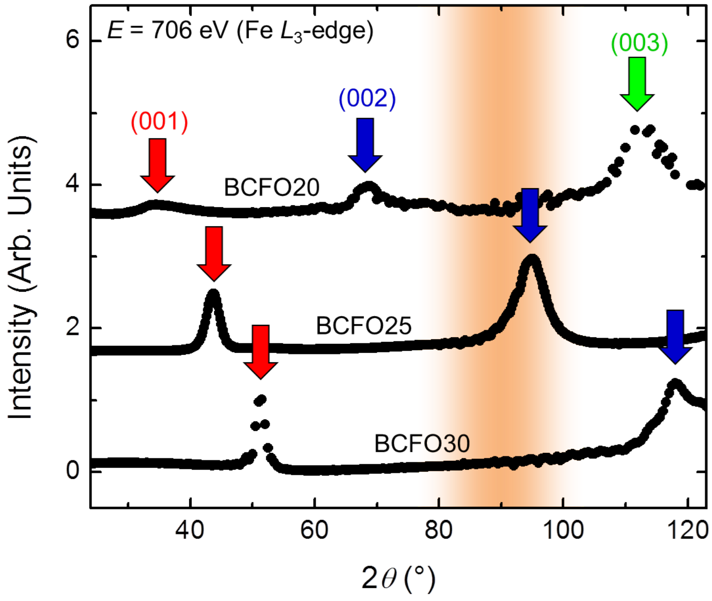

Figure S1. Manipulating the periodicity of the oxygen vacancy ordering by Ca^{2+} substitution ratio. First, second, and third superstructure reflections are indicated by red, blue, and green colored arrows, respectively. The intensities are divided by Q^4 and shifted for clarity, where Q is the absolute value of momentum transfer in unit of $Å^{-1}$. Orange area near 2θ = 90 ° indicates the vicinity of Brewster's angle.

[S. 2] Brewster's angle and suppression of structural signal at 2θ = 90° and selection of (002) reflection of BCFO25

When a light strikes a surface, the amount of reflection and transmission is determined by refractive index of the target material (n), incident angle (θ_i), and polarization rate of the light. Geometry of incident and scattered light is shown in Fig. S2a. At a certain incident angle, no π polarized light is reflected. The angle is called as Brewster's angle ($\theta_B = 90° - \theta_i$) and determined by $\tan\theta_B = n/n_0$, where n_0 is a refractive index of vacuum[S2]. In x-ray region, n is very close to 1, and therefore θ_B is nearly identical to 45° (Fig. S2b).

In general, intensity of Thomson scattering which is from structural contribution is determined by $(\varepsilon_i \cdot \varepsilon_f)$ where ε_i (ε_f) is polarization of incident (scattered) light[S3]. Since we only control a polarization of the incident x-ray polarization, the scattered photon's polarization always shows both σ_f and π_f polarizations. In this sense, when we use the π as the ε_i, the term, $\pi_i \cdot \sigma_f$, becomes zero. This is because an angle between two polarizations' directions is orthogonal. Therefore, the structural contribution is determined solely by $\pi_i \cdot \pi_f$ in the π channel. When $2\theta = \theta_i + \theta_f =$ 90° (Fig. S2b), $\pi_i \cdot \pi_f$ also becomes zero. Consequently, with π polarized light, there is little structural contribution from either reflection or diffraction near $2\theta =$ 90° (e.g. BCFO25 (002) reflection as shown in Fig. S1). Furthermore, we need to consider an addition polarization term in the resonant x-ray scattering, i.e., $(\varepsilon_i \times \varepsilon_f)$[S3], showing the 2×2 polarization matrix. Diagonal terms in the matrix are proportional to isotropic form factor (i.e.,

structural factor), which is similar effect in $(\varepsilon_i \cdot \varepsilon_f)$. Off-diagonal terms in the matrix are sensitive to detect anisotropic contributions such as spin, orbital, and charge disproportion.

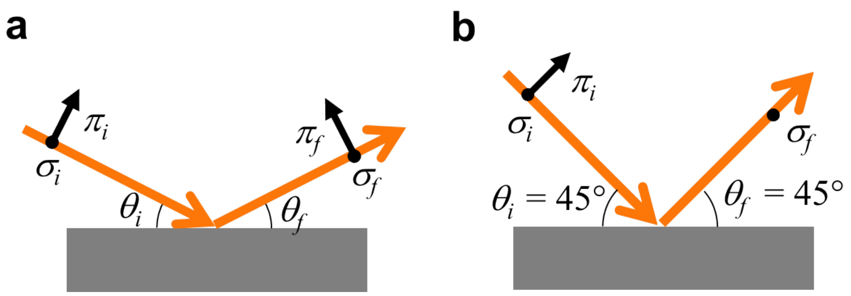

Figure S2. (a) Simple description of incident and scattered x-ray geometry with incident (θ_i) and scattered angle (θ_f) with linear polarization (σ,π). (b) At Brewster's angle position ($\theta_i = \theta_f = 45°$), no structural contribution from π polarized light.

[S. 3] Subtraction of diffuse scattering

Even in diffraction condition, unexpected signals can be detected as well as pure diffraction signal. One of them is fluorescence background. It is photon energy dependent but there is little angle dependence. Therefore, if we subtract a signal from out of diffraction condition, we can remove fluorescence background.

Fig. S3a shows rocking (θ) scan of BCFO25 (002) reflection. Figure S3b and S3c show RSXS profiles at both on diffraction condition (red colored arrow in Fig. S3a) and

diffuse condition conditions (blue colored arrow in Fig. S3a) of (002) in incident σ polarization and π polarization, respectively. σ_i RSXS profile contains complicated structural modification by oxygen vacancy (Fig. S3b). In π_i, structural contribution is suppressed, and therefore nearly pure electronic signal can be obtained (Fig. 4b in main article). (001) reflection which is away from Brewster's angle show complicated structural contribution in both σ_i and π_i cases (Fig. S4).

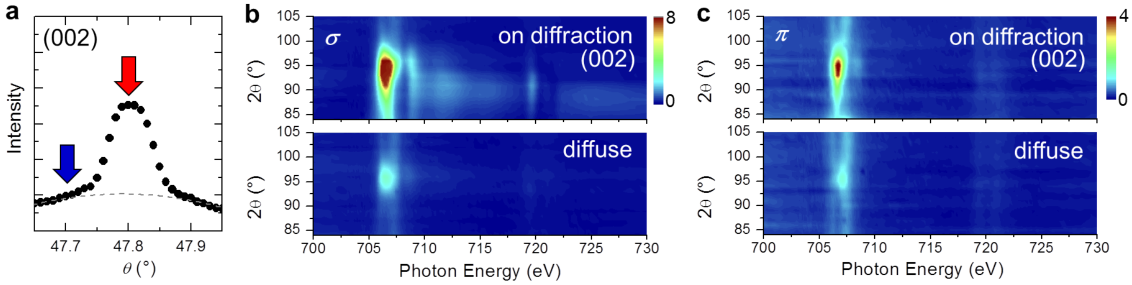

Figure S3. (a) Red and blue colored arrow indicate the position of on diffraction and diffuse conditions of (002) reflection. RSXS profiles on diffraction condition and diffuse condition of (b) (002) with σ_i and (c) (002) with π_i. Complicated structural contribution is suppressed with π_i in contrast to structural contribution dominant σ_i case.

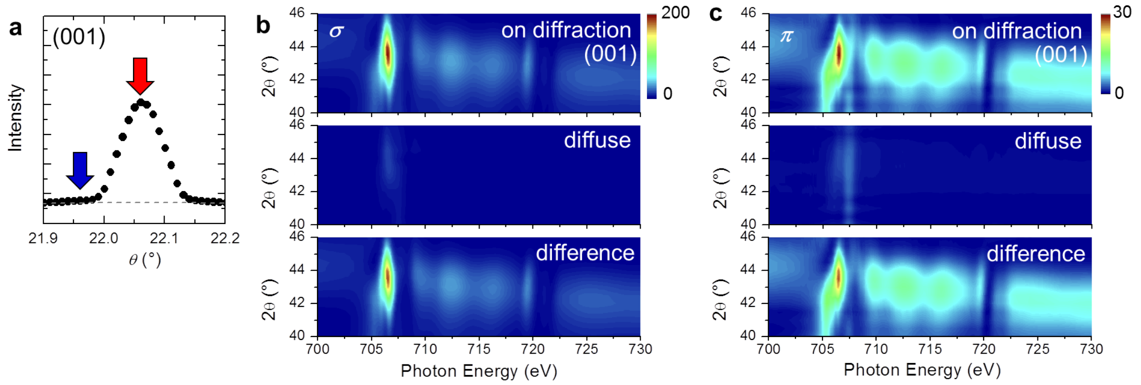

Figure S4. (a) Red and blue colored arrow indicate the position of on diffraction and diffuse conditions of (001) reflection. RSXS profiles at on diffraction condition, diffuse condition, and their difference of (b) (001) with σ_i and (c) (001) with π_i

REFERENCES

(S1) Yang, C.-H.; Seidel, J.; Kim, S. Y.; Rossen, P. B.; Yu, P.; Gajek, M.; Chu, Y. H.; Martin, L. W.; Holcomb, M. B.; He, Q.; Maksymovych, P.; Balke, N.; Kalinin, S. V.; Baddorf, A. P.; Basu, S. R.; Scullin, M. L.; Ramesh, R. *Nat. Mater.* **2009**, *8*, 485–493.

(S2) Brewster, D. *Philos. Trans. R. Soc. Lond.* **1815**, *105*, 125–159.

(S3) Hill, J. P.; McMorrow, D. F. *Acta Crystallogr. A* **1996**, *52*, 236–244.
